# Optimization of *Ralstonia solanacearum* cell growth using a central composite rotational design for the P(3HB) production: Effect of agitation and aeration

**DOI:** 10.1371/journal.pone.0211211

**Published:** 2019-01-29

**Authors:** Mariane Igansi Alves, Karine Laste Macagnan, Camila Rios Piecha, Matheus Marques Torres, Izadora Almeida Perez, Sônia Maria Kesserlingh, Rosane da Silva Rodrigues, Patrícia Diaz de Oliveira, Angelita da Silveira Moreira

**Affiliations:** 1 Department of Food Science and Technology, Eliseu Maciel Faculty of Agronomy, Federal University of Pelotas, Pelotas, Rio Grande do Sul, Brazil; 2 Technological Development Center, Biotechnology, Federal University of Pelotas, Pelotas, Rio Grande do Sul, Brazil; 3 Center for Chemical, Pharmaceutical and Food Science, University Federal of Pelotas, Pelotas, Rio Grande do Sul, Brazil; 4 Company PHB Industrial, Serrana–SP, Brazil; Tallinn University of Technology, ESTONIA

## Abstract

The intracellular accumulation of polyhydroxyalkanoates (PHAs) normally occurs after cell growth, during the second fermentation stage and under nutrient-limited conditions in the presence of a carbon excess. However, some microorganisms are able to accumulate PHAs as poly(3-hydroxybutyrate) [P(3HB)] during the first fermentation stage, the cell growth phase, without nutrient limitation, once they have been reported to utilize type II metabolism during the polymer accumulation phase. This study evaluated the effect of aeration and agitation on cell growth and P(3HB) accumulation in *Ralstonia solanacearum* RS, performed in a bioreactor for 24h at 32°C. A 2^2^ central composite rotational design (CCRD) was used, with agitation (150 to 250 rpm) and aeration (0.3 to 1 vvm) as independent variables and optical density (OD_600nm_), dry cell weight (DCW), and P(3HB) yield as dependent variables. A significant polymer accumulation, until 70% of P(3HB), was observed, proving that *R*. *solanacearum* RS exhibited metabolism type II, regardless of the aeration process. The best results were obtained for 1 vvm and 250 rpm (+1, +1), with values of OD_600nm_ (18.04) and DCW (4.82 g.L^-1^).

## Introduction

In the last six decades, 8.3 billion metric tons of plastics have been produced, most of which disposable single-use plastics [1]. It is estimated that 91% of all plastics produced is not recycled, and 6.3 billion metric tons has become plastic waste [1,2]. For years, scientists have been investigated ways to reduce these numbers to prevent the volume of plastics that end up in the world's oceans, causing damage to marine mammals, birds, and fish, among others. By the middle of the century, there will be more plastics in the oceans than fish––a chilling prognosis [2]. In this context, the use biodegradable polymers may be an effective alternative to reduce the excessive amount of plastic waste in the environment, thus leading to a lower environmental impact [3,4].

Studies on the use of polymers have been carried out to offer products with good mechanical properties and a less drastic impact on the environment, including the biodegradable polymers, which can reduce the plastic pollution.

Polyhydroxyalkanoates (PHAs) are among the biodegradable polymers that can be used as substitutes for polymers from petrochemical resources, and the poly(3-hydroxybutyrate) [P(3HB)] has been highlighted [5]. The great attraction of these biomaterials is that they are fully biodegradable and non-toxic, and can be produced from renewable sources [6,7]. Although P(3HB) has many advantages when compared with plastics made from petroleum, its commercialization is still quite limited due to the high cost of production, thus, studies of cost savings are required. Microbiological processes can substantially reduce the production cost by using more affordable substrates and optimization of the fermentation process [8].

Over 300 microorganisms can synthesize PHAs; however, the production of PHAs is usually limited to *Ralstonia* spp., *Cupriavidus necator*, *Pseudomonas* spp. and recombinant *Escherichia coli* [6, 9–11]. *Ralstonia* spp. tend to be more amenable to production on an industrial scale demonstrating high yields and production rates [12] and accumulating approximately 80% of their dry weight in polymers [13].

The strain used in the present study, *R*. *solanacearum* RS, is a phytopathogenic bacterial P(3HB) producer, which was isolated from a cactus in the state of Rio Grande do Sul, southern Brazil, and characterized by 16S rRNA sequencing [14, 15]. *R*. *solanacearum* RS was selected for the study, due to the novelty of studying this species for the production of intracellular biopolymers, such as P(3HB)s.

Preliminary studies conducted by our research group using orbital shaker incubators found that *R*. *solanacearum* RS began its log phase of growth from 6 h of incubation, entering the stationary phase at 22 h. Glucose and sucrose were effective as carbon sources, and sucrose was the most appropriate source for cell growth, and the acidic pH assisted in a higher polymer yield, as a function of the carbon source used (sucrose or glucose). Thus, the microorganism was classified as having type II metabolism, once it did not require nutrient restrictions for the biopolymer accumulation [16].

Although several parameters can be used in the fermentation process, which is usually changed one by one, this strategy does not allow studying the interaction among different parameters, therefore, the process response is given by only one variable. However, the fermentation behavior is influenced by several factors, thus the statistical optimization including the temperature, pH, agitation rate, and aeration should consider the interaction between the variables in the generation of a process response [17] significant role in P(3HB) accumulation in various bacterial strains using inexpensive carbon sources [18].

In this study, the use of a 2^2^ central composite rotational design (CCRD) [19, 20] has shown that studying these variables is extremely important to obtain effective results in the production of biopolymers.

The goal of this study was to evaluate the effect of aeration and agitation on cell growth and accumulation of P(3HB) in *R*. *solanacearum* RS, using a 2^2^ central composite rotational design (CCRD). The experiment was performed in a bioreactor, aiming at a greater cell growth used a new concept to monitoring cell growth in the P(3HB) production of combine de methods OD_600nm_ and DCW in the inoculum phase.

## Materials and methods

### Microorganism

The *R*. *solanacearum* RS strain was purchased from the Laboratory of Bacteriology of the Eliseu Maciel Faculty of Agronomy, Federal University of Pelotas, RS, Brazil. The microorganism was freeze-dried and stored at -80°C, and sub-cultured monthly on nutritive yeast agar [21] composed of (in g.L^-1^) peptone (Kasvi), 5.0; glucose (Synth), 5.0; yeast extract (Kasvi), 1.0; meat extract (Himedia), 3.0; and agar (Kasvi), 15.0, and then stored at 4°C.

### Culture media and operating conditions

The pre-inoculum was formed by the suspension of fresh cells, obtained from multiplicative cultures, grown in NYA solid medium plates [21] for 48 h at 32°C, with an initial cellular concentration (OD_600nm_) of 0.5. The absorbance was determined in a spectrophotometer (Ultrospec10, United Kingdom). Volumes of 200 mL were placed into 500 mL Erlenmeyer flasks. The processes were conducted in an orbital shaker incubator with a yeast malt [22] culture medium composed of (in g.L^-1^) yeast extract (Kasvi), 2.7; malt extract (Kasvi), 2.7; peptone (Kasvi), 4.5; sucrose (Synth), 40. The conditions were 32°C, pH 6 and 250 rpm for 24 h.

Growth curves were performed to compare the results of cellular growth (OD_600nm_) in the inoculum phase from the bioreactor (Biostat B, Germany) and those from the shaker (Certomat BS-1, Germany). The experiment conditions in the shaker were the same of the pre-inoculum phase, while the conditions in the bioreactor were 32°C, 250 rpm, 1 vvm, and pH 6. Samples were collected every two hours for 48 h. DCW yield, P(3HB) accumulation, and sugar and nitrogen residuals throughout the fermentation process were measured only in the bioreactor.

To determine the effect of the agitation and aeration on the inoculum production, a bioreactor with a capacity of 10 L and useable of 7 L, pH 6, with initial cellular concentration (OD_600nm_) of 0.5, at 32°C for 24 h was used. A complete factorial design 2^2^ with 3 levels (-1, 0, +1) was used, with four treatments at the axial points and three at the central point, totaling 11 treatments (Table 1). The independent variables were aeration (0.3 to 1 vvm) and agitation (150 to 250 rpm).

**Table 1:** Matrix of the 2^2^ CCRD. Coded and real levels of aeration and agitation and the response variables of the *R*. *solanacearum* RS inoculum incubated for 24 h at 32°C in YM culture medium, using sucrose as the carbon source.

Treataments	Independent variables			Dependent variables			
	Aeration (vvm)	Agitation (rpm)	pH	OD_600nm_	DCW (g.L^-1^)	P(3HB) (%)	Productivity from the DCW (g.L^-1^.h^-1^)
1	-1 (0.3)	-1 (150)	6.3	8.61	2.13	26.5	0.08
2	+1 (1)	-1 (150)	5.7	15.31	4.0	39.65	0.15
3	-1 (0.3)	+1 (250)	5.8	15.37	4.81	36.62	0.19
4	+1 (1)	+1 (250)	5.9	18.04	4.82	42.87	0.19
5	-1.41 (0.25)	0 (200)	6.3	11.73	2.00	23.11	0.07
6	+1.41 (1.05)	0 (200)	6.5	13.24	1.93	45.06	0.06
7	0 (0.65)	-1.41 (143)	6.3	10.82	2.85	43.98	0.10
8	0 (0.65)	+1.41 (257)	5.8	13.99	4.33	39.94	0.17
9	0 (0.65)	0 (200)	5.7	16.09	4.51	31.76	0.17
10	0 (0.65)	0 (200)	5.7	16.40	4.50	31.17	0.17
11	0 (0.65)	0 (200)	5.7	15.44	4.57	31.28	0.18

pH, potential of hydrogen; OD_600nm_, optical density; DCW, dry cell weight; P(3HB), poly(3-hydroxybutyrate).

All experiments were carried out in triplicate. Comparisons were analyzed by ANOVA using the Statistica 7.0 program, considering p < 0.05 as significant.

The Response Surface Methodology (RSM) was used to determine the best processing conditions (in terms of aeration and agitation) to increase the cellular multiplication of *R*. *solanacearum* RS.

### Cell growth and P(3HB) accumulation

The optical density (OD_600nm_) was measured in a spectrophotometer at 600 nm to determine the cell growth.

The dry cell weight concentration (DCW) and P(3HB) accumulation were determined by gravimetry. First, 50 mL of fermented broth was centrifuged (10.000 × g for 15 min). Then, the cell concentrates were resuspended in 0.89% (w/v) saline solution and centrifuged again. The DCW was obtained by drying the pellets in an oven at 56°C until constant weight [16].

The P(3HB) accumulation was determined by chemical extraction using a method that allows recovery up to 98% of accumulated polymer[23]. To calculate the accumulation yield, the samples were weighed, and the results were expressed as a percentage, according to Eq 1.

%Y=(P1÷P2)×100 (1)

where P1 is total weight of the recovered bioplastics, and P2 is DCW.

The P(3HB) productivity was calculated as the concentration of P(3HB) produced per hour whereas P(3HB). All averages were calculated from triplicate measurements.

### Total residual sugars

To evaluate the amount of carbon source consumed, sucrose residuals (a non-reducing sugar) from the cultures were determined by the dinitrosalicylic acid (DNS) method for reducing sugars [24]. Samples from centrifuged fermented broth were hydrolyzed with 2 M HCl and then neutralized with 2 M NaOH. The supernatants were diluted to 1:40 or 1:50 (v.v^-1^) as needed. The procedures were done in test tubes with 1 mL of sample added to 1 mL of the DNS reagent. The samples were stirred and heated at 100°C for 5 min, and then placed in a cooling bath for 5 min, and 16 mL of sodium and potassium double tartrate solution was added. The absorbance readings were carried out in a spectrophotometer (HITACHI, model: U-1800; Japan) at 540 nm. A standard curve of glucose ranging from 0 to 1.0 g.L^-1^ was constructed, and the results were expressed as g.L^-1^.

### Total residual nitrogen

To determine the amount of nitrogen source consumed, a Urea CE (Ref. 27) commercial test (Labtest, Brazil) was used to evaluate the residual nitrogen. The analyses were carried out in accordance with the Labtest instructions. The samples used were supernatants of fermentation broths obtained from determination of P(3HB) accumulation. The results were expressed as g.L^-1^.

### Gel Permeation Chromatography (GPC)

The molar mass of the polymers was estimated by GPC analysis. The assay was developed by analysts at PHB Industrial SA (Serrana, SP, Brazil) and is not commercially available. The following Waters GPC equipment and accessories were used: 1515 isocratic pump, column heater, 717 plus autosampler, 2414 refractive index detector, and Styragel columns (10^3^, 10^4^, 10^5^, and 10^6^; 7.80 x 300 mm). The samples were solubilized in chloroform, filtered through a 0.45 μm membrane, and spiked with toluene as a peak marker prior to injection. The mobile phase was HPLC analytical grade chloroform.

## Results and discussion

**Fig 1:**
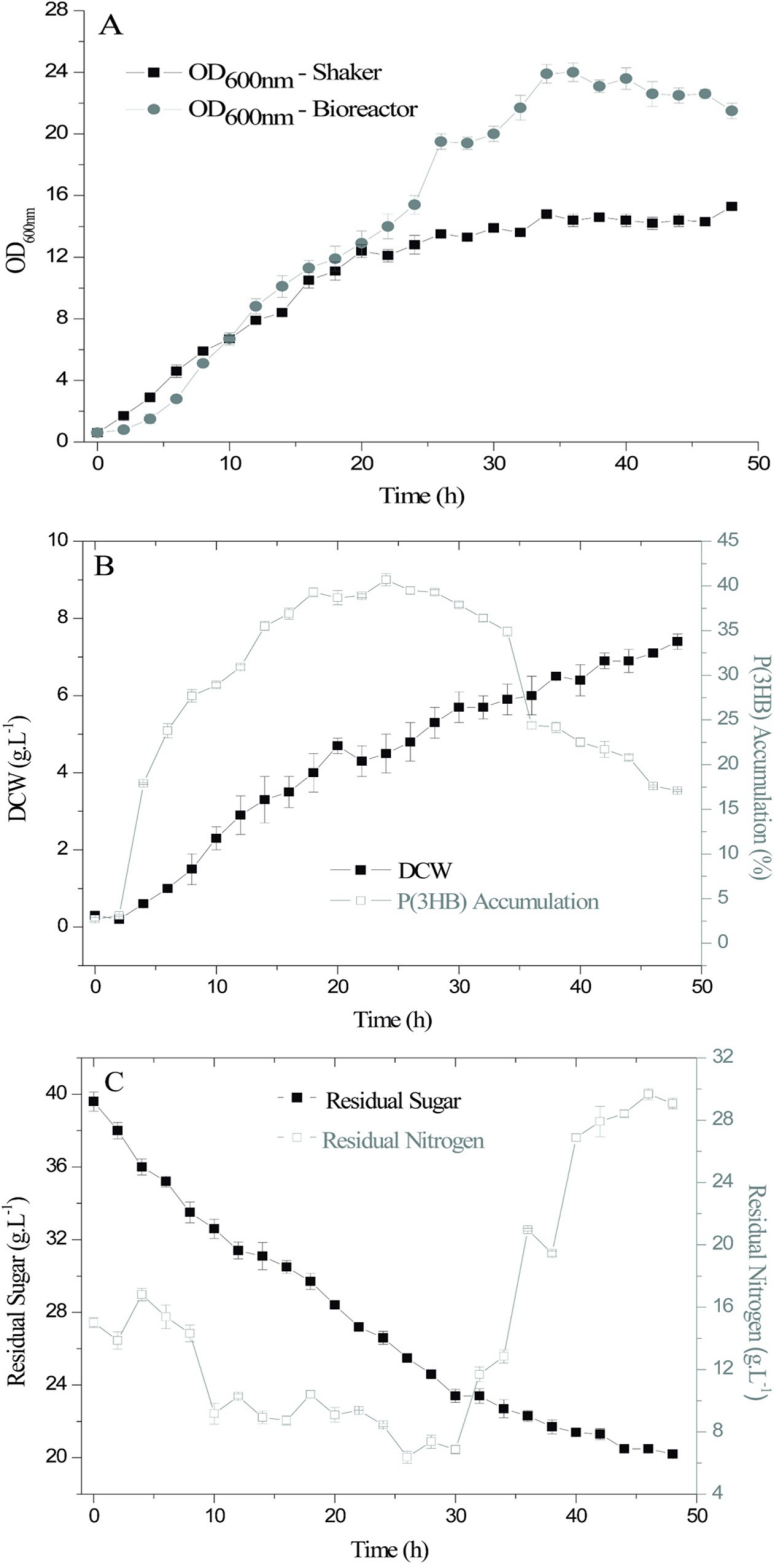
Growth curves of the *R*. *solanacearum* RS strains, P(3HB) accumulation and sugar and nitrogen residuals. (A) shaker and bioreactor, cellular concentration expressed as OD_600nm_; (B) bioreactor, dry cellular weight (DCW) and P(3HB) accumulation (g.L^-1^); (C) bioreactor, sugar and nitrogen residuals during 48 h (g.L^-1^).

The growth curves in terms of OD_600nm_ of the *R*. *solanacearum* RS strains developed in both the shaker and the bioreactor are shown in (Fig 1A, 1B and 1C).

As shown in Fig 1A, the growth curves obtained in both apparatus and expressed as OD_600nm_ were equivalent until 20 h. After 26 h of process, significant differences were observed, with higher results for the fermentation in the bioreactor when compared to the shaker. The highest cell growth values expressed as OD_600nm_ were found at 34 h (23.9 abs) in the bioreactor and (14.8 abs) in the shaker. The P(3HB) productivity was 0.12 g.L^-1^.h^-1^ in the bioreactor. Cultures with high cell densities have advantages such as a higher concentration of product, and higher productivity and recovery costs of polymer [25]. These positive results are due to the conditions in the bioreactor, which provides a controlled environment that allows for efficient cell growth and attainment of the product of interest, which is not observed in the shaker, as it is not possible to control the agitation conditions.

Similar behavior was observed for the growth curves, which exhibited a lag phase within 5 h, followed by the log phase up to 34 h, without entering the stationary phase. The decline phase in turn cannot be observed. Macagnan et al. (2017) [16] reported that the stationary phase of the growth curve of *R*. *solanacearum* RS in YM medium [22], also expressed as OD_600nm_, was within 22 to 28 h of culturing.

Microbial growth curve is an important parameter in microbial research, and is a complex process, which depends on numerous coordinated anabolic and catabolic reactions. The analysis of cell growth can be performed using both the discontinuous and continuous modes [26], and the discontinuous growth curve was used in the present study. The discontinuous process is characterized by the use of known concentrations of nutrients, with no input or output of any nutrient during the period of analysis. Thereby, the amount of nutrients decreases and the rate of metabolite residues increase [26]. In the continuous growth, the flow of nutrients supplied to the microorganism remains the same, and despite the cells increase in size, the microbial growth is mainly due to binary division [27,28].

For the bioreactor (other parameters were not analyzed for the shaker), DCW increased over time, with a greater yield of 7.6 g.L^-1^ (48 h). During the bioprocess, the amount of biomass is an important physiological parameter related to cell growth, metabolism, and productivity, along with the OD_600nm_ and other parameters offline conventional biomass in this type of process [29,30]. Although the OD_600nm_ was practically stabilized within 36–48 h, great changes were observed in DCW and P(3HB) accumulation. When the cell characteristics change during growth, including the intracellular polymer concentration, the relationship between OD_600nm_ and DCW also changes. The resulting inaccuracy is sometimes inconsequential, but becomes important when one of the primary variables of interest is the biomass yield itself. Then, the OD_600nm_ measurements used alone can transmit little information on cell concentration [31], making it necessary to combine methods for better monitoring of the inoculum phase, such as OD_600nm_ and DCW.

In the growth curve, a plateau relative to P(3HB) recovery from 16 h (36.9%) to 28 h (39.3%) (Fig 1B) was verified. Thus, a time of 24 h was established for the realization of the 2^2^ CCRD, because a decrease in yield is observed when the DCW values were related to the P(3HB) yield, even at high OD_600nm_ and DCW values, with the greatest accumulation within 24 h (41.32%). An increase in DCW with the decrease in P(3HB) is indicative of high cell multiplication and polymer degradation to supply energy and carbon, as the metabolism of the microorganisms is governed by the growth medium. With a balanced growth, the microorganism uses the substrate as an energy source and/or for the maintenance/formation of cellular material [32, 33].

As expected, the sugar concentration decreased during the process, corresponding to half of the initial concentration available at the end of the process (Fig 1C). These results show that the microorganisms began to use the polymer produced for survival, rather than all sugar available, resulting in the low polymer accumulation at the end of the growth curve. The availability of the carbon source in the medium should also be regarded as a positive factor for the polymer accumulation as the cultures examined for P(3HB) yield contained approximately 50% of the initial sucrose level after 48 h of incubation. However, higher sugar concentrations can hamper cell multiplication and the early P(3HB) accumulation, as reported by Crochemore et al. (2012) [34].

The nitrogen concentration decreased within 30 h (from 0.15 g.L^-1^ to 0.6 g.L^-1^), and increased after this period, reaching 0.29 g.L^-1^ at the end of the process, within 48 h. This increase may be due to the cell degradation, once microbial cells are composed of nitrogenous bases. For the production of P(3HB), it is usually necessary to limit the essential nutrients [35] such as nitrogen, as high values of this micronutrient eventually suppress the polymer formation, which can be noticed by the growth curve. Ramsay et al. (1990) [12] reported that the nitrogen concentrations below 0.2 g.L^-1^ allowed for the P(3HB) accumulation by *R*. *eutropha*.

PHA producing microorganisms are divided into two groups, I and II. Group I comprises those microorganisms that accumulate the biopolymer only under special conditions, i.e. with an excess of carbon and the nutrient restriction, such as N, P, Mg, K, S or O. In these microorganisms, the cellular growth and polymer accumulation occur in different phases. On the other hand, group II consists of microorganisms with no nutrient restriction or carbon excess [36]. Although the *Ralstonia* spp. lineage is generally classified as belonging to group I [36–38], Macagnan et al. (2017) [16] reported a P(3HB) production of 46.62% in the inoculum phase, without nutrient restriction, which is a characteristic of group II. Crochemore et al. (2012) [34] described extensive P(3HB) accumulation—up to 70%, by the *Pseudomonas* strain CMM43 in the inoculum phase, suggesting that the accumulation occurred simultaneously with growth.

### 2^2^ central composite rotational design

Table 1 presents the results of cell growth (OD_600nm_ and DCW) and P(3HB) accumulation as a function of the different aeration and agitation conditions, following a central composite rotational design (2^2^ CCRD).

Table 2 presents the results of analysis the variance (ANOVA) of the P(3HB) yield according to the central composite rotational design (2^2^ CCRD).

**Table 2:** Analysis of variance (ANOVA) for the quadratic polynomial model fitted for the maximum P(3HB) yield. Analysis of variance (ANOVA) of aeration and agitation of the response variables of the *R*. *solanacearum* RS inoculum incubated for 24 h at 32°C in YM culture medium using sucrose as the carbon source.

Source of variation	FD	Sum of squares	Mean square	F	Pr > F
Model^a^	5	472.5702	94.51404	5.5	5.05
Error	5	68.1097	13.62194	-	-
Lack of fit	3	67.9128	22.6376	19.164	19.164
Pure Error	2	0.1969	0.09845	-	-
Total	10	540.6799	-	-	-

FD: Freedom degree

^a^R^2^ = R^2^ = 0.87403, R^2^_ajusted_ = 0.87403

Table 3 presents polydispersity data and the average Mm for recovered P(3HB) from treatments as a function of different aeration conditions and agitation of the culture medium, generated by the application of the Central Composite Rotational Design (CCRD 2^2^).

**Table 3:** Polydispersity index and average Mm for the recovered P(3HB). Gel Permeation Chromatography (GPC) of the aeration and agitation treatment for the *R*. *solanacearum* RS inoculum incubated for 24 h at 32°C in YM culture medium using sucrose as the carbon source.

Treatments	Polydispersity (Mm/Mn)	Average Molar Mass (Da)	
		Mn	Mm
1	2.14	3.5 × 10^5^	7.5 × 10^5^
2	4.03	8.7 × 10^4^	3.5 × 10^5^
3	4.17	3.7 × 10^4^	1.5 × 10^5^
4	2.98	1.1 × 10^5^	3.4 × 10^5^
5	2.07	2.8 × 10^5^	5.8 × 10^5^
6	3.84	1.4 × 10^5^	5.4 × 10^5^
7	4.32	1.1 × 10^5^	5.0 × 10^5^
8	5.30	7.5 × 10^4^	3.9 × 10^5^
9	5.38	6.8 × 10^4^	3.6 × 10^5^
10	5.67	3.9 × 10^4^	2.2 × 10^5^
11	6.00	5.2 × 10^4^	3.1 × 10^5^

Mn, Number average molecular weight; Mm, Weight average molecular weight

The initial pH of the medium was 6.0, which was not adjusted during the process. It was observed that lower pH reductions were associated with lower DCW values. In the treatment 6 (+1.41; 0) the final pH (6.5) was associated with the highest P(3HB) accumulation.

The highest cell growth estimated by OD_600nm_ (18.04) and DCW (4.82 g.L^-1^) was observed in the treatment 4, which combined +1; +1, and reproduced the growth curve conditions. The results showed that greater agitation, even with lower aeration, can result in a satisfactory density and cell mass at the maximum value, but when related to the low supply of oxygen in the minimum values, it ends up having a lower production of the polymer. In the mathematical model tested, the dependent variables OD_600nm_ and DCW did not have predictive and significant results, thus it was not possible to generate response surfaces. Macagnan et al. (2017) [16] evaluated the pH conditions in the inoculum phase to optimize the production process of P(3HB) by *R*. *solanacearum* RS, and found that a more acidic pH may indicate higher cell growth.

The treatments 5 (-1.41; 0), 6 (+1.41; 0), and 7 (0; -1.41), with OD_600nm_ values of 11.73, 13.24 and 10.82, obtained lower DCW values of 2 g.L^-1^, 1.93 g.L^-1^, and 2.85 g.L^-1^, respectively. Among these treatments, the treatment 6 presented the highest OD_600nm_ and the lowest DCW. Therefore, it is important to study other parameter, such as DCW, in association with the optical density to obtain a satisfactory and real process characterization.

Significant P(3HB) accumulation was verified in all treatments, confirming that *R*. *solanacearum* RS should be classified as type II in relation to the polymer accumulation phase. As observed for the DCW, the P(3HB) yield was remarkable in the treatment 4 (42.87%), which combined +1; +1. On the other hand, the treatment 6, with aeration and agitation at levels +1.41; 0, respectively, had lower OD_600nm_ and DCW values, and the highest P(3HB) yield (45.06%). The productivity values varied from 0.08 g.L^-1^.h^-1^ to 0.19 g.L^-1^.h^-1^, corresponding to the treatments 1 and 4, respectively. A predictive mathematical model with 95% confidence and determination coefficients (R^2^) of 0.87 (Table 2) for the dependent variable of P(3HB) was generated. The coefficient of determination (R^2^) measures the proportion of the total response variation that is explained by the model. Models with R^2^ *<*0.60 should be used only as trend indicators and never for predictive purposes [39–41].

The statistical analysis showed that 95% of significant regression coefficients were considered in the mathematical models (Eq 2) proposed to represent P(3HB) accumulation as a function of the aeration and agitation processes.

$$P(3HB)=31.41+6.31\times Ae+0.93\times {Ae}^{2}+0.96\times Ag+4.89\times {Ag}^{2}-1.73\times Ae\times Ag$$ (2)

where, Ae is the variable aeration and Ag is agitation.

**Fig 2:**
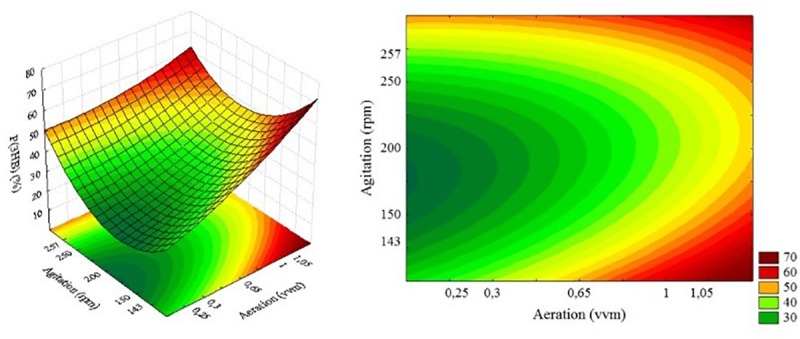
(A) Response and contour surfaces as a function of P(3HB) accumulation for the variables aeration and agitation.

The response and boundary surface for the aeration and agitation, based on the model generated by Eq 2, are shown in Fig 2.

The results of the response surface in relation to the P(3HB) production shows that higher biopolymer production (about 70% higher) tends to occur with a greater availability of oxygen.

Eq 2 was used to perform the validation of the response surface generated by the P(3HB) production, replacing the variables Ae (aeration) and Ag (agitation) by the coded variables +1 for both aeration and agitation, which should produce 42.77% P(3HB) yield, similar to the 41.32% obtained experimentally, indicating that the model proposed can be reproduced.

In relation to the polydispersity index (Mn/Mm), the highest values were observed for the treatments 8 (5.30), 9 (5.38), and 11 (6.0), with aeration and agitation at the levels 0; +1.41; and 0; 0, respectively, with values ranging from 1.5 ×10^5^ Da (T3) to 7.5 × 10^5^ Da (T1). The average molar mass (Mm) of P(3HB) produced by *R*. *solanacearum* RS strain was 2.9 × 10^5^ Da [23]. This property is extremely important because it directly affects the mechanical strength of the bioplastics as well as the expansion capacity, the hydrolysis, and consequently, the biodegradation rate [42].

Kemavongse et al. (2008) [43] investigated the poly-β-hydroxyalkanote production by halotolerant *Rhodobacter sphaeroides* U7, using aeration values from 0 to 1.5 vvm and agitation values from 0 to 200 rpm. The two best results, DCW of 5.08 g.L^-1^ and P(3HB) yield of 57.84%, were obtained by medium aeration and high agitation (1 vvm and 200 rpm) and high aeration and agitation (1.5 vvm and 200 rpm) respectively.

Chakraborty et al (2012) [44] studied the PHA production and yield of *R*. *eutropha* fed intermittently with short chain fatty acids, using a mean OD_600nm_ of 1.04 abs for inoculum conditions, and a culture medium described as CCS (solubilized corn), which is a low cost industry medium, for 24 h at 30°C and 250 rpm. The PHA productivity (0.0697 g.L^*−*1^.h^*−*1^), PHA (8.37 g.L^*−*1^), and PHA content (39.52%) were high when ARF (artificial rumen fluid) was fed every 3 h for 61 h. However, the productivity varied by 0.19 g.L^-1^.h^-1^ in 24 h, which was lower than that observed in the present study.

Zafar et al. (2012) [45] investigated the modeling and optimization of the poly(3-hydroxybutyrate-co-hydroxyvalerate) production from cane molasses using a bioreactor, and the effect of agitation and aeration regime on *Azohydromonas lata* MTCC 2311 inoculum, at 180 rpm and 30°C for 24 h. The authors reported a higher P(3HB-co-3HV) productivity of 0.163 g.L^*−*1^.h^*−*1^, which is similar to the findings of the present study. However, only the inoculum production conditions can be compared with the current study.

Macagnan et al (2017) [16] used a central composite rotational design (2^2^ CCRD) aimed at adjusting the pH and sugar concentration in the inoculum phase of *R*. *solanacearum* RS in a shaker. In the optimized range, the authors found DCW and P(3HB) accumulation of 5.35 g.L^-1^ and 45.62%, respectively. When compared with the present results, it is evident that the biopolymer accumulation can exhibit the highest yield above the maximum aeration conditions, regardless of agitation in the bioreactor.

The factorial design is a continuous practice and has become an important tool, capable of determining better the processing conditions, with advantages when compared to the conventional methods using only one parameter per test. A number of researchers have used the factorial technique to optimize the culture conditions [46,47] and to determine the optimum processing parameters [48,49].

## Conclusion

In growth curves, OD_600nm_ exhibited similar values at 20 h in both the shaker and bioreactor, with the highest OD_600nm_ observed in the bioreactor. Higher DCW values (7.6 g.L^-1^) and lower P(3HB) yield were observed over time. The sugar residuals decreased during the process, and nitrogen also decreased within 30 h, increasing afterwards. According to the present results, the best aeration and agitation conditions in the inoculum phase was 1 vvm and 250 rpm (pH 5.9, OD_600nm_ 18.04, DCW 4.82 g.L^-1^, and 42.87% yield P(3HB). In relation to the P(3HB) accumulation by *R*. *solanacearum* RS in the initial phase, it can be said that the microorganism belongs to group II, once it has no metabolic requirements for the biopolymer production. Further studies should be performed to evaluate whether the growth and P(3HB) accumulation in the microorganism studied can be improved by a continuous approach.
